# A proposed model using glycation metrics and circulating biomarkers for the prevention of cardiovascular disease

**DOI:** 10.3389/fmed.2025.1624682

**Published:** 2025-08-12

**Authors:** Timothy Valk, Carol McMorrow

**Affiliations:** CardiacData Analytics, Winter Park, FL, United States

**Keywords:** glycation, biomarkers, algorithm, cardiovascular disease, prevention

## Abstract

**Introduction:**

Cardiovascular aging starts early in life due to the glycation of critical proteins, though its progression remains undetected in the formative years. The glycation reaction affects all tissues by the same non enzymatic irreversible reaction. The variables are the pH, temperature, glucose concentration, and the specific protein. This relationship implies that glycated blood biomarkers could potentially be used as a proxy for assessing *in situ* myocardial changes.

**Methods:**

Laboratory tests for troponin I (cTnI), hemoglobin A1c (A1c), fructosamine, and low-density lipoprotein (LDL), were chosen to calculate the proxy for *in situ* glycation. An algorithm was developed incorporating these variables as individual measurements and as calculated metrics of glycation. This data was obtained from previous large group studies of variables and outcomes.

**Results:**

Modeling of glycation was determined for each variable. Using metrics from multiple studies, theoretical rates of glycation of LDL and troponin I were calculated. The glycated changes in LDL and troponin I were used to determine the increases above optimal physiological rates.

**Conclusion:**

Laboratory results of LDL, cTnI, A1c and fructosamine could be used sequentially to derive a cost-effective proxy for assessing *in situ* aging and deterioration of cardiovascular tissue. This model could theoretically predict the rate of cardiovascular aging by integrating four blood biomarkers into a dedicated algorithm guiding proactive diagnostics and treatment.

## 1 Introduction

In 2021 cardiovascular disease caused over 21 million deaths worldwide, which is one third of all deaths globally. While this is commonly thought to be a disease of developed countries it is now known that over three quarters of the deaths are in low- and middle-income countries. Ischemic heart disease (IHD), specifically, stands as the leading cause of premature death in 146 countries for men and 98 countries for women (1). While the classic myocardial infarction (MI) symptoms of chest pain and shortness of breath are often mentioned, they are in the minority of cases. Seventy to 80% of transient episodes of cardiac ischemia are not associated with any symptoms (2). In asymptomatic middle-aged adults, 12.5% had evidence of silent myocardial ischemia when actively monitored during normal activity (3). Additional data suggests 20–60% of all myocardial infarctions are silent (4, 5). For decades IHD progresses in adults without obvious symptoms. Frequently the initial presentation is cardiac arrest which affects over 350,000 individuals per year in the USA (6). The ability to determine the progression of heart disease is a significant challenge. Algorithms such as the Framingham heart model which began in 1948 have limited use as the risk is only based on a 10-year projection (7). Of those in the lowest risk quartile, 58% have subclinical and 36% significant atherosclerotic cardiovascular disease (8). Artificial intelligence (AI) rare data predictive modeling improves the diagnosis of heart disease but requires analysis of unstable or abnormal cardiac and metabolic factors (9). Present diagnostic methods require CT, MRI or PET scans, stress tests or cardiac catheterization to determine clinical heart disease; however they have difficulty in predicting it during the silent phase of development. A multinational study of 13,540 adults using 4,963 plasma protein concentrations showed only modest improvement when polygenic risk score was added to standard risk evaluation (10). The polygenic data analysis required complex and expensive testing of 60 genetic variants. Certain population groups have a significantly greater risk of silent heart disease. Women often do not develop the classic symptoms of ischemia and are therefore underdiagnosed. In women with IHD, only 30% have chest pain as a prodrome for a MI (11, 12). Minority populations have greater rate of heart disease and cardiac mortality due to a marked prevalence of hypertension, diabetes, lipid disorders and metabolic syndrome (13). For decades the standard of care has been to evaluate apparently healthy individuals using resting electrocardiogram (EKG), lipid concentrations, blood pressure, blood glucose, smoking and family history and then decide a plan of action. However, this approach cannot determine the rate of cardiovascular deterioration. There are two overlooked facts about heart disease. (1) If an algorithm estimates a 20% risk of a cardiac event within 10 years, it has predicted that one of five identical individuals will develop significant heart disease, but it cannot determine which specific individual will be affected. (2) The reported data on cardiac risk is error prone due to the significant number of silent events that are not detected. Present diagnostic evaluations are expensive, time consuming, and frequently invasive. They also only determine cardiac disease once it is clinically evident. Frequent testing by these methods is not feasible. Using the USA as an example, over 10,000,000 cardiac stress tests are done yearly at cost of an average of $1,000 per test (14, 15). Over 40% of all Medicare part B medical imaging expenditures each year are spent on nuclear cardiac stress tests, a cost of $17 billion annually (16). An estimated 32–48% of all stress tests done each year may not be needed (17). This does not include costs of outpatient care, hospitalizations, heart catheterizations and medications. The cost of cardiovascular disease in the USA alone for 2020 was greater than $300 billion and estimated to increase to over $1 trillion by 2050 (18). The costs worldwide are difficult to estimate (1). To sequentially determine the progression of asymptomatic heart disease would require a testing methodology that is cost effective, noninvasive, automated and correlates with *in situ* cardiovascular aging. The glycation reaction plays a crucial role in triggering the catabolic cascade, making its understanding essential for developing the proposed testing system.

## 2 Methods

### 2.1 Risk analysis and present algorithms

The described algorithm is based on analysis from available data in numerous studies. It does not use original data. Risk analysis for LDL, troponin, A1c and fructosamine has been detailed in multiple large studies from one or more countries. The data reveals increased risk of cardiac events with increase in each analyte concentration over a fixed period of time (19–23). These are discussed in the sections on “glycation.” However, the proposed model does not evaluate group risk. Standard analysis of group risk compares changes in a control group and comparative group over time but cannot evaluate each individual. An analogous test to this model is dual energy X-ray absorptiometry (DEXA) testing of bone density (24). Bone density is measured by DEXA scanning and represented as age related (Z-score) and young adult comparison (T-score) scores. While group risk analysis can be determined, the evaluation of each individual is based on their previous and present results. Treatment options are based on each individual’s density changes and their specific situation. Risk analysis and prediction patterns may not be useful as individuals have unique patterns of change and variable treatment options.

Present algorithms for cardiovascular disease have inadequate predictive abilities. The sensitivities and specificities are 69%/62% for the Framingham model (FRS-CVD), 34%/ 85% for the European CVD model (SCORE) and 46%/82% for the Scottish Heart Health model (ASSIGN). These models do not use biomarkers of glycation or A1c in their calculations. Only the ASSIGN model uses diabetic status (25).

### 2.2 Glycation overview

Glycation is caused by the contact of glucose with a protein, lipid or nucleic acid and adducts to the substrate. The variables in this reaction are temperature, pH, glucose concentration and the physical characteristics of the substrate. These physical characteristics include half-life, turnover rate and molecular structure. It is an irreversible reaction with an early phase of Schiff base and later phase of Amadori product and advanced glycation end products (AGE) formation. While the early phase may be linear, the overall reaction is complex and probably has nonlinear characteristics (26). We have postulated linear kinetics for the initial conceptual modeling as non-enzymatic reactions are primarily linear (27). Further studies will be required to clarify the actual kinetics. Glycation initiates a cascade of factors which accelerate aging in the body (28). The final result is the production of AGE. AGE are inflammatory compounds which form even during normoglycemia. However, dysglycemia increases the rate of reaction in a pathological manner. The specifics of glycation in cardiovascular tissues and circulating biomarkers are discussed in the next sections.

### 2.3 Glycation of cardiovascular tissue

**Figure 1:**
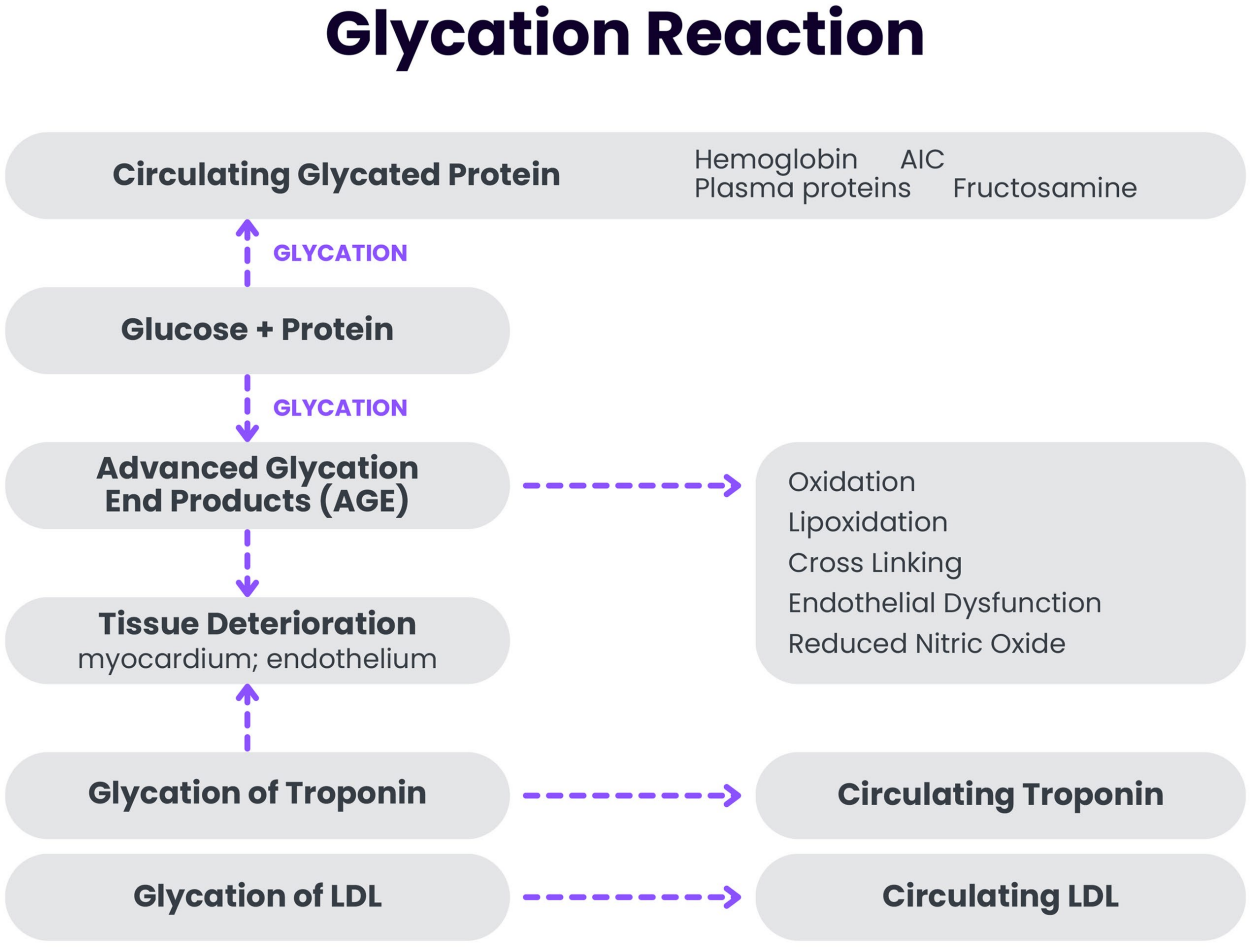
Schematic drawing of the glycation reaction.

The AGE produced by glycation have catabolic effects on the myocardium and vascular endothelium as noted in Figure 1. AGE induce protein cross-linking with increased trapping of low-density lipoprotein (LDL) in the arterial wall (29, 30). They also reduce protective nitric oxide production causing endothelial dysfunction (31) and accelerate telomere attrition by inducing inflammatory mediators (32). AGE have been causally related to oxidation and lipooxidation in the pathogenesis of atherosclerosis. AGE produced by glycation amplify reactive oxidation. The deleterious effects of AGE have been correlated with coronary artery disease and cardiac event risk. Myocardial turnover is only 1% per year at age 25 declining to 0.45% per year at age 75 (33–39). Because myocardial tissue has a low turnover rate, even a marginal increase in blood glucose concentrations and the resultant AGE production would have a significant detrimental effect over a lifetime. Myocardial troponin is used as the critical biomarker of cardiovascular tissue deterioration in this model.

### 2.4 Glycation of circulating biomarkers

#### 2.4.1 Hemoglobin A1c (A1c) and fructosamine

Glycation occurs in blood biomarkers with the same reaction dynamics as in cardiovascular tissues. The two most commonly glycated blood proteins measured in clinical practice are A1c and fructosamine, the later consisting of plasma proteins primarily albumin (40–44). The glycation rate of circulating proteins is related to their exposed lysine residues (45). There is an inverse relationship between the number of lysine residues which bind glucose and the substrate half-life. The normal percent of hemoglobin glycated is 4–6% while fructosamine is 10–15%. The half-life of fructosamine is proportionally shorter than A1c (46). The result is an equivalent amount in glycation of these blood proteins (47, 48). The risk of developing heart disease is positively correlated with A1c, fructosamine and AGE (49–51). Increasing A1c concentrations correlate with increases in AGE (52–55). An increase in A1c from 39 to 46 mmol/mol (5.7–6.4%) correlates with a change in AGE of 48% (56). Early diastolic echocardiographic deterioration correlates with increase in AGE and A1c in the prediabetic range (57). Deterioration in cardiac, lipid, and glucose metabolism has been shown to correlate with increasing A1c in prediabetic individuals (58).

#### 2.4.2 Troponin I (cTnI)

Contraction of the heart is controlled by the enzymatic protein troponin which has 3 subunits. Troponin I (cTnI) is the subunit which controls the relaxation phase (59, 60). Troponin I concentrations have a greater accuracy than troponin T in determining mortality risk in the general population (61, 62). The presently used highly sensitive troponin I assay can detect a measurable quantity in the blood of >99% of healthy adults (63). Troponin I increases from age 20 and has been shown to be a predictive biomarker of silent heart disease in healthy adults (64–66). It has also been shown to be correlated with cardiac mortality independent of the number of obstructive coronary artery lesions as well as in those without significant lesions (67). Troponin I concentrations in adults without heart disease between ages 40–60 were persistently lower in women than in men but with women exhibiting a relatively larger increase with advancing age. The median change in cTnI concentration was a 4.4% increase per year in women and a 3.5% increase per year in men beginning at age 45 (68). Receiver Operating Characteristic (ROC) optimal cut off in adults for diagnostic accuracy of heart disease was determined to be 5.1–5.2 ng/ L regardless of number of coronary arteries involved. One study was done in the USA on a mixed population while the other was done in the UK predominately in men. This illustrates a common range in dissimilar groups (67, 69). Some studies have suggested different sex related troponin risk concentrations (70) with a ROC cut off for women of 4.5–4.7 ng/L and men 5–7 ng/L (65, 67, 69). This difference is postulated to be in part from the smaller left ventricular size in women (71). At a median age 54 years, 13% of a healthy cohort (10% of women and 20% of men) had cTnI >5 ng/L. At a median age 62 years, 25% of a healthy cohort (15% of women and 35% of men) had cTnI of > 5 ng/L (72). Of those age 18–29, 9% had troponin concentrations above the upper limits of normal (73). In those <40 years of age, a majority of the abnormalities were from conditions such as myocarditis, pulmonary embolism and cardiomyopathy (74, 75). The presence of troponin in the blood of healthy adults can be related to multiple myocardial cellular functions but even concentrations in the normal range for age appear to be from subclinical myocardial necrosis (39, 76, 77). The effects of glycation and resultant AGE production cause irreversible modifications to the structure and function of all troponin subunits (78, 79).

#### 2.4.3 Low-density lipoprotein (LDL)

Native LDL is relatively inactive until glycated under the same conditions as for other proteins. It then initiates the cascade of catabolic and inflammatory effects by producing AGE. The AGE induce oxidation, foam cell formation, and endothelial dysfunction with reduction of nitric oxide. The AGE also increase vascular permeability, procoagulant activity, and atherogenesis (80–84). LDL concentrations are positively correlated with the development of atherosclerotic cardiovascular disease and mortality from < 100 mg/dL to >190 mg/dL (85). The process is markedly reduced at 60–80 mg/dL (86).

### 2.5 Modeling of biomarkers and biomarker metrics

#### 2.5.1 Introduction

The information presented illustrates the effects of glycation on proteins *in situ* and in blood. The integration of the blood biomarkers into metrics which act as a proxy for the in-situ process is the focus of this article. In the modeling derivation, certain assumptions are used.(1) A1c concentrations in venous and capillary blood are highly correlated with a Pearson correlation coefficient (r) > 0.94 equating glucose concentrations in venous and myocardial blood (87). Therefore, myocardial troponin is exposed to the same blood glucose concentrations as circulating hemoglobin and plasma protein. (2) The irreversible non enzymatic reaction for glycation of troponin I and LDL is the same as for hemoglobin A1c and fructosamine. It is characterized by half-life, turnover rate, structure and concentration of each variable (26). This was discussed in the previous section on “glycation.” (3) Glycation is a physiologic process and has a rate which allows for optimal tissue function. It cannot be modified without intervention. Increased rates of glycation are pathological (88). (4) The rate of glycation for an individual can be expressed proportionally to the calculated optimal glycation rate of troponin I and LDL and expressed as a ratio exceeding that level. This is discussed in the next section. (5) There is a steady state between concentrations of troponin I released from the myocardium and in the peripheral circulation of healthy adults. An increase in blood troponin I is proportional to the amount released from cardiac tissue (60). (6) Protein/lipid/nucleic acid structures of an individual are genetically coded and do not change. Therefore, the glycation of a specific individual substrate is not altered over time due to molecular modification (89). (7) Glycation of LDL is an essential mechanism in the pathogenesis of atherosclerosis and cardiovascular injury: LDL is relatively inactive without glycation (80–84). (8) The assay of circulating troponin I uses a highly specific monoclonal antibody to native troponin I. This suggests the molecular structure of circulating and *in situ* troponin I are equivalent (90) and would have the same glycation.

The eight assumptions above are referenced but the data available is limited. These are assumptions and additional data and analysis are necessary to verify their accuracy.

The next sections explain the model used as a proxy for in situ glycation and aging of the myocardium and vascular endothelium.

#### 2.5.2 Mathematical model: variable physical characteristics

The variables in the irreversible glycation reaction in humans are (1) pH (2) temperature (3) duration (4) the glucose concentration which can be determined by hemoglobin A1c and fructosamine, and (5) the protein substrate. The protein substrate is characterized by the concentration, half-life, and molecular structure. In the healthy adult, pH and temperature are relatively constant at 7.35–7.45 and 37°C, respectively. The duration of the reaction is the life span of the individual. Over a lifetime, the continuous reaction in each healthy individual is assumed to be at physiological pH and temperature (91, 92). The half-lives of each protein have been given constant values based on known data. Half-lives of hemoglobin A1c and fructosamine are 28.7 and 16.5 days, respectively (93). These are stable in healthy adults absent of interfering conditions (94, 95). Using the same constants for pH, temperature, and half-lives of hemoglobin A1c and fructosamine allow for individual sequential analysis. Troponin I has a half-life calculated as short as 2–4 h and as long as 3.2 days dependent on ischemic or stable conditions (96–98). In this model a half-life constant of 1 day has been used in all cTnI calculations. The half-life of LDL has been determined to be 2–4 days and in these calculations 3 days has been used as the half-life constant (99, 100). These could be adjusted within the formulas for specific subsets and indications.

#### 2.5.3 Mathematical model: formula calculations

Glycation is a physiological reaction which has an optimal rate. Using A1c as a reference, the optimum is approximated at 31 mmol/mol (5.0%). Values above and below 31 mmol/ mol have an increasing cardiac mortality risk (101). While glycation could be reduced by lowering the glucose concentration to an A1c < 31 mmol/mol, the available glucose for critical metabolic functions would also be reduced. There is a linear correlation between A1c and fructosamine. A fructosamine concentration of 200 umol/L has been calculated to be equivalent to an A1c of 31 mmol/mol (102). This value is also used in the calculations as the optimal glycation rate of plasma protein. Calculation of the relative increase in glycation for an individual is based on the optimal value of 1.0 and expressed as a ratio. Calculation of a glycation rate requires the use of the slope-intercept equation with the formula m = (Y2 − Y1)/(X2 − X1); b = Y1 − m∗X1 (103). The half-lives of A1c, fructosamine, LDL and troponin I (28.7, 16.5, 3, and 1 day(s), respectively) are the X variables (93–100). The ratios of individual glycation rates to the optimal are the Y variables. The following example illustrates the calculations for an individual with an A1c of 39 mmol/L (5.7%) and fructosamine of 240 umol/L.

Calculation for relative troponin I glycation rate (TGR)

A1c 5.7% (39 mmol/mol)/5.0 (31 mmol/mol) = 126% (1.26).

Fructosamine 240 umol/L/200 umol/L = 120% (1.20).

Plotting the slope-intercept formula:

(A1c) X_1_ = 28.7, Y_1_ = 1.26.
(fructosamine) X_2_ = 16.5, Y_2_ = 1.20.
(troponin I) X_3_ = 1: Y_3_ =?

Y_3_ = m(X_3_) + b.

m = 0.0049 b = 1.12.

Y_3_ = troponin glycation rate (TGR) = 1.12 (12% above optimum).

Calculation for relative LDL glycation rate (LGR).

(A1c) X_1_ = 28.7, Y_1_ = 1.26.
(fructosamine) X_2_ = 16.5, Y_2_ = 1.20.
(LDL) X_3_ = 3, Y_3_ =?

Y_3_ = m(X_3_) + b.

m = 0.0049 b = 1.12.

Y_3_ = LDL glycation rate (LGR) = 1.13 (13% above optimum).

#### 2.5.4 Mathematical model: biomarker metrics

- Troponin glycation rate (TGR): the relative rate of glycation for troponin I expressed as a ratio to the optimum of 1.0.
- Troponin glycation index (TGI): the calculation of the total troponin glycated cTnI × TGR = TGI.
- LDL glycation rate (LGR): the relative rate of glycation for LDL expressed as a ratio to the optimum of 1.0.
- LDL glycation index (LGI): the calculation of the total LDL glycated LDL × LGR = LGI.

#### 2.5.5 Mathematical model: calculated range of TGI and LGI

1. Glycated troponin (TGI)
  - Lowest troponin glycation index (TGI) is A1c 31 mmol/mol and fructosamine 200 umol/L (relative value of 1.0) X troponin I 1.6 ng/L [limit of detection] (104) = 1.0 × 1.6 = 1.6.
  - Highest troponin index (TGI) is 48 mmol/mol and fructosamine 285 umol/L (relative value of 1.5) X troponin I 4.5 ng/L for women = 6.75 and 1.5 × 5 ng/L for men = 7.5.
2. Glycated LDL (LGI)
  - Lowest LDL glycation index is A1c 31 mmol/mol and fructosamine 200 umol/L (relative value of 1.0) X LDL 60 mg/dL = 1.0 × 60 = 60.
  - Highest LDL glycation index is A1c 48 mmol/mol and/ructosamine 285 umol/L (relative value of 1.5) × 180 = 270.
3. Limiting and exclusionary factors: A1c > 46 mmol/mol (>6.4%) suggests diabetes mellitus (105): LDL > 180 mg/dL suggests severe hyperlipidemia (106): troponin I > 4.5 ng/L for women and > 5 ng/L for men suggests cardiac ischemia or injury (65, 67, 69). These are the exclusionary limits used for the above calculations but can be modified.

Calculated ranges for:

TGI: 1.6–6.75 (women): 1.6–7.5 (men).

LGI: 80–270.

TGR and LGR 1.0–1.5.

Quartile ranges in Table 1 are determined from the above data. The upper limit cTnI concentration of exclusion for men could use values between 5–7 ng/L (65, 67, 69, 70, 107) by adjusting the ROC curve. The present calculations have used 5 ng/L to improve sensitivity.

**Table 1:** Quartiles for biomarkers and metrics.

Quartiles	Troponin I (ng/L)	Troponin glycation rate (TGR)	Troponin glycation index (TGI)	LDL (mg/dl)	LDL glycation rate (LGR)	LDL glycation index (LGI)
1	1.6–2.4^a^	1.0–1.15	1.6–2.8	60–90^d^	1.0–1.15	60–104
2	2.5–3.3	1.16–1.31	2.9–4.3	91–121	1.16–1.31	105–160
3	3.4–4.5/5.0^b^	1.32–1.50	4.4–6.75/7.5^c^	122–152	1.32–1.50	161–228
4	>4.5: female^b^	>1.50^e^	>6.75^c^	153–180	>1.50^e^	229–270
4	>5.0: male^b^	>1.50^e^	>7.5^c^	153–180	>1.50^e^	229–270

Upper allowable limits for the calculations are: LDL: 180 mg/dl. A1c: 48 mmol/mol (6.5%). Fructosamine: 285 umol/L. Troponin I: 4.5 ng/L for females and 5 ng/L for males. ^a^1.6 ng/L is the limit of detection (LOD) for troponin I assay (104). ^b^ROC risk cut off for females >4.5 ng/L and males >5 ng/L (65, 67, 69). ^c^TGI >6.75 for females: > 7.5 for males. ^d^60–80 mg/dL is concentration limiting atherosclerosis development (86). ^e^>1.5 requires exclusionary value of A1c > 48 mmol/mol (>6.5%).

## 3 Application of the model to evaluate a simulated healthy adult

**Figure 2:**
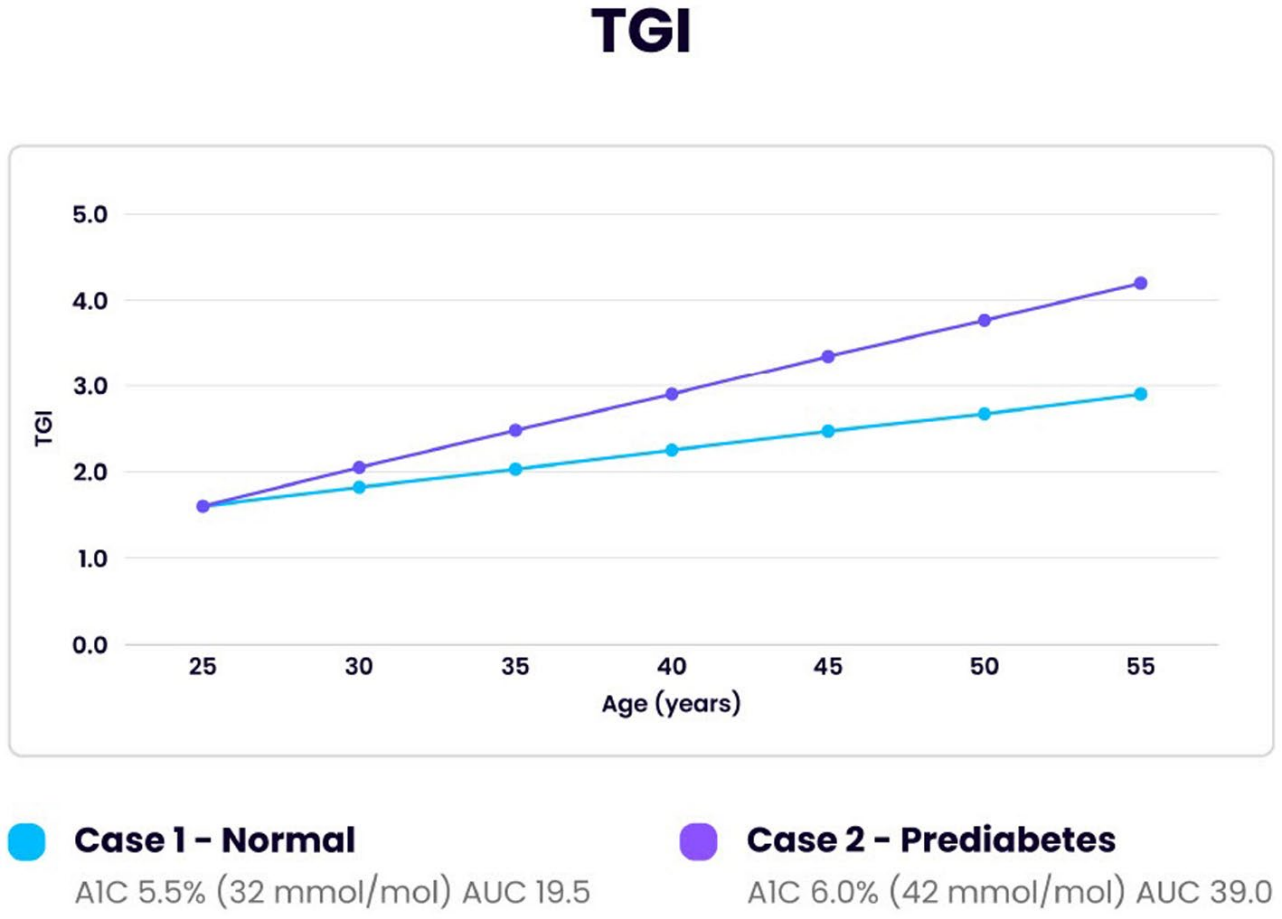
Comparison of Troponin I glycation index (TGI) over 30 years for one individual developing prediabetes and one normal individual. Initial reference point at age 25 for both was with the same optimal reference TGI of 1.6, A1c 31 mmol/mol (5.0%), TGR 1.0 and cTnI 1.6 ng/L. Area under the curve (AUC) revealed a doubling of the amount of myocardial tissue glycated in the prediabetic individual compared to the normal (39/19.5).

**Figure 3:**
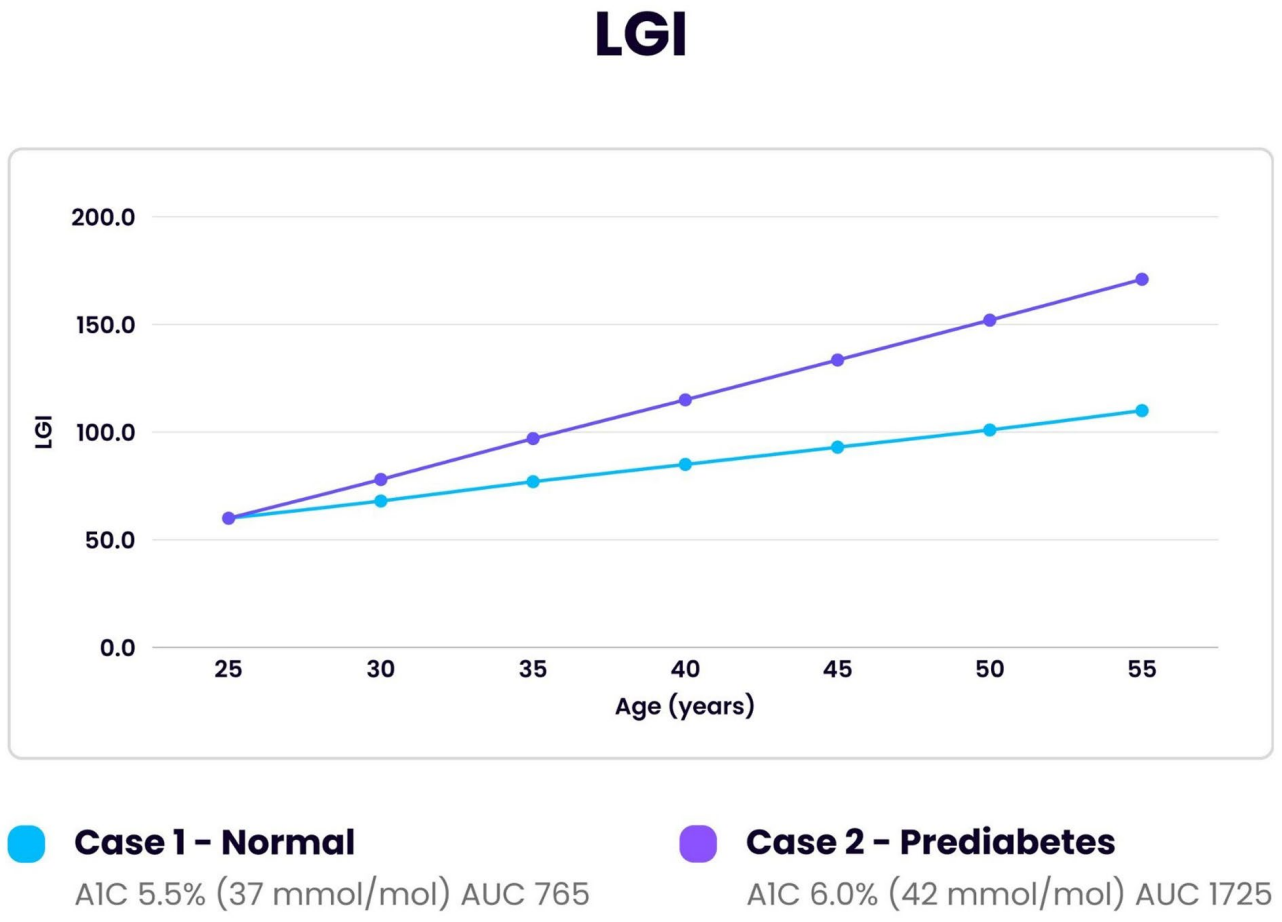
Comparison of LDL glycation index (LGI) over 30 years in an individual developing prediabetes and one normal individual. Initial reference point at age 25 for both was LGI 60, A1c 31 mmol/mol (5.0%), LGR 1.0 and LDL 60 mg/dL. The area under the curve (AUC) for the individual with prediabetes demonstrated greater than twice the amount of LDL glycated compared to the normal individual (1725/765).

- Algorithm analysis and report: the results of the algorithm calculations are shown in this section. The accuracy of this algorithm is dependent on the repetitive use of monitoring from age 20–30 to age 60. It requires sequential annual use or proposed timeline. A single determination is inadequate. Infrequent and delayed monitoring can lead to inaccuracies.
  1. Table 1 illustrates quartiles for biomarkers and metrics as calculated from the data noted in the previous section. These limits can be adjusted as needed. An example would be the lower limit of detection (LOD) for cTnI. If the assay improves and has a lower LOD the new value could be substituted. In addition, the upper limit of cTnI for exclusion could be adjusted independently to adjust the specificity and sensitivity. Quartiles could also be adjusted for specific data analysis.
  2. Table 2 illustrates a spreadsheet for a simulated individual at annual intervals for 4 years. The 4 biomarker and 4 calculated metrics are entered in the specified columns. The predicted values for the next year (2026) are calculated by a linear regression equation (108). In this example the individual progresses from normal to prediabetic classification as determined by A1c (105). This data can be formatted into a final report using Table 1.
  3. Figure 2 illustrates glycation of troponin I (TGI) in two simulated individuals. One pattern demonstrates a normal individual while the other an individual who develops prediabetes. The baseline point of reference for both is the optimum value of 1.6 as noted in the previous section. Over 30 years the normal and the prediabetic individuals have increased TGR from 1.0 to 1.11 and 1.0 to 1.24, respectively. In the same amount of time the cTnI value for the normal individual increased to 2.6 ng/L and the prediabetic individual to 3.4 ng/L. The greater TGR in the prediabetic individual produced a proportionally higher cTnI (44). The prediabetic individual produced twice the amount of glycated troponin I (TGI) over 30 years calculated by area under the curve (AUC) (109). This would cause increased myocardial aging.
  4. Figure 3 illustrates glycation of LDL (LGI) over a 30-year span using the same data as for the previous TGI calculation. The LGR for the normal individual increased from 1.0 to 1.11 and for the prediabetic individual from 1.0 to 1.25. The LDL increased from 60 mg/dL to 100 mg/dL and from 60 mg/dL to 140 mg/dL in the normal and the prediabetic individuals, respectively. Altered metabolic effects in prediabetic individuals cause proportionately higher LDL concentrations (110). The changes noted in the prediabetic individual produced more than a doubling in the amount of LDL glycated (LGI) compared to the normal. The doubling of LDL and troponin I glycated in individuals with prediabetes increases the rate in deterioration of cardiovascular tissue. This is significant as prediabetes affects 38–46% of the general population (111, 112).
- Sequential changes in biomarker and metric patterns.

**Table 2:** Representative report.

DATE	A1c (%)	A1c mmol/mol	Fructosamine (umol/L)	Troponin I (ng/L) cTnI	Troponin glycation rate (TGR)	Troponin glycation index (TGI) TGR X cTnI	LDL (mg/dl)	LDL glycation rate (LGR)	LDL glycation index (LGI) LGR X LDL
2022	5.5	37	230	2.6	1.1	2.9	110	1.1	121
2023	5.5	37	230	3.0	1.1	3.3	120	1.1	132
2024	5.7	39	240	3.2	1.12	3.6	125	1.14	143
2025	6.0	42	262	3.4	1.26	4.3	130	1.27	165
2026^a^	6.1	43	267	3.7	1.25	4.6	137	1.33	176

Representative annual report for biomarkers and metrics of a simulated individual. ^a^Represents predicted 2026 results.

Table 2 illustrates an example of an increase in cTnI concentrations over a 4-year period. Group data has shown an increase of cTnI over longer periods of time (68, 70). This was well documented in the large MORGAM/BiomarCaRE study of individuals from ages 30–60 (65). Circulating troponin in healthy adults represents release into the blood because of marginal ischemia or remodeling with the circulating concentration proportional to the release rate (39, 76, 77, 79, 113). Evaluation of the biomarkers and their metrics could determine possible causes of changes of cTnI. A parallel increase of TGR with cTnI increase would suggest glycation as the cause of the change: if TGR did not increase at a proportional rate then other factors such as hypertension could be a cause (74). Declines in cTnI concentrations can occur with medications such as statins (69). Statins lower cTnI and LDL, but do not lower glucose (114). The lowering of LDL by statins reduces the substrate of the glycation reaction. This would reduce AGE production, and the troponin release would decline. This pattern was seen in the SPRINT trial correlating improved blood pressure control with lowered troponin concentrations and cardiac risk independent of lipids or glucose (114, 115). Lifestyle changes such as diet and exercise also can lower LDL, troponin and glycation (116–118). Each individual would have a biomarker pattern dependent on their own lifestyle, genetics, and metabolism. Glycation is a major determinant in the biomarker variations. The age-related increase in cTnI is due in part to the parallel increase in A1c and resultant glycation. The prevalence of prediabetes with A1c 39–46 mmol/mol has increased 3-fold in adolescents ages 12–19 between 1999–2020 (119, 120). This creates a greater degree of glycation before 20 years of age (120, 121). The effect of glycation is further magnified by LDL which increases in men by 64% from age 20–49 and in women by 42% from age 35–59 (122). The increase in these biomarkers and their metrics with advancing age (61–63, 69) adds to the complexity of the sequential patterns. Because of the individual variability of the biomarkers and metrics due to age, health and medication adjustments, predictive analysis over the long term would be difficult and often inaccurate.

## 4 Discussion

Cardiovascular disease is the most common cause of death in both men and women in the USA. The mortality rate exceeds that of cancer and accidents combined (123). While treatment advances have reduced the mortality rate over the last 25 years it has continued to be the most common cause of death (124). Glycation is a non-enzymatic irreversible reaction which has a significant impact on cardiovascular disease. It occurs universally with any adduct of a carbohydrate and a substrate of protein, lipid or nucleic acid (26). It was originally described by a French chemist and chef Louis-Camille Maillard in 1912 during food preparation (125) and has been used in diverse applications. The physical properties of the substrate as well as the carbohydrate concentration are primary factors in determining the glycation rate (26, 27). Glycation in humans by glucose is an integral determinant of aging (28, 32, 126). The effect on tissue is permanent: only regeneration or replacement can repair it. The myocardium has a minimal turnover and repair rate of 1% or less per year (35–39). Therefore, critical myocardial tissues are significantly affected by marginal increases in glycation over a lifetime due to the production of catabolic and inflammatory AGE. Each individual has a unique genetic, environmental and metabolic signature (88, 127). As a result, the analysis of group data has not been shown to be effective in reducing cardiac risk (7, 128, 129). A dedicated algorithm is needed to calculate the changes in glycation rate using an individual’s sequential data. Specific blood biomarkers can be used to calculate the needed data by acting as a proxy for the in-situ process. Glycation of hemoglobin (A1c) and plasma protein (fructosamine) have the same reaction determinants as troponin I and LDL (27–30). Previous sections detailed the specifics involved in the modeling of the algorithm using the results of available blood tests. The specific laboratory tests required (troponin I, fructosamine, A1c and lipid profile with direct LDL) are available at commercial laboratories for a modest cost. The Center for Medicare and Medicaid Services (CMS) reimbursement rate for the 4 laboratory tests in 2024 was $54.08 (130). These rates are used as guidelines for commercial insurance. The prevalence of heart disease before age 40 is 0.9% (131) which allows the initial values to be used as a baseline for an individual during the years of heart disease progression. Those with initial values outside the targeted range as determined by the provider or guidelines would be referred for evaluation. Increased rates of glycation can counter genetically coded protective mechanisms such as collateral circulation and altered AGE binding on receptors (RAGE) (132–134). The effects of glycation can be demonstrated visually in the aging of skin and connective tissues due to loss of elasticity and irreversible cross linking of collagen. These AGE-induced effects correlate with the rate of aging in cardiovascular tissue (135–139). Software integrating data from multiple organs and tissues could develop an algorithm of human aging. The effects of glycation are magnified by the long reaction time. As seen in Figures 2, 3 a doubling in the amount of cTnI and LDL glycated can occur over 30 years with only a modest increase in A1c from 37 to 42 mmol/mol (5.5–6.0%). An increase in A1c from 39 to 46 mmol/mol (5.7–6.4%) correlates with an increase in AGE of 48% (56). While the glycation reaction can be slowed it is continuous and irreversible (26). Therefore, it is essential to begin sequential testing in early adulthood. Data from the United Kingdom (UK) National Health Service (NHS) shows increase in primary care cost per individual of $450 and hospital cost of $5,000 in the year following a myocardial infarction. This does not include cost of medications or invasive procedures. The 7.6 million living in the UK with heart disease have twice the annual cost of care (140). Preventative care with the laboratory tests and modeling discussed could significantly reduce costs and mortality. Another potential use of the algorithm could be in clinical drug trials as an objective measure comparing placebo and treatment groups. Glucagon-like peptide 1(GLP -1) drugs have been approved for use in individuals with significant heart failure, sleep apnea, or heart disease in type 2 diabetes (141–144). They have been proposed to extend life expectancy (145). Clinical trials of these and other drugs comparing placebo and treatment groups of healthy adults using the data from the algorithm could lead to new treatments in the prevention of heart disease and related conditions. The comparative data could be used to define an endpoint in addition to risk reduction. Groups divided by sex, age and ethnicity could evaluate specific outcomes for each subgroup. The development of new drugs could advance the treatment of those most affected by this silent disease. IHD has wide range in prevalence-based factors such as ethnicity, genetics and race (13). It is especially prevalent in African American women where it affects 47% (146, 147). The use of the developed algorithm could significantly reduce cardiovascular disease in these populations.

## 5 Limitations

Active smokers have a greater risk of heart disease but have lower troponin I concentrations (148). This could cause erroneous results. Unstable glucose concentrations due to rapid weight gain/ loss or recent medication change could produce discrepancy in A1c and fructosamine correlation and lead to inaccuracies. This could be evaluated by calculating the glycation gap which determines glucose stability (149, 150). Intercurrent conditions such as anemia, as well as acute and chronic illnesses can alter the test results. The testing should be done under direction of medical professionals after history and physical examination supported with routine laboratory tests such as complete blood count (CBC) and comprehensive metabolic profile (CMP). The needed frequency of testing is unknown. Annual testing is postulated to concur with an annual medical examination. More frequent testing could be done to determine changes with additive treatment. Tests results which seem inaccurate or unusual should be repeated. However, the frequency of studies would need to be based on data and consensus. Accurate analysis requires sequential data on a regular timeline such as at annual examinations. It also requires initiation of testing in early adulthood. The ages and intervals for testing would be at the direction of the medical provider and with guidelines from professional associations. Laboratory analysis should be done using the same methodology. Troponin I measurements are often done on different equipment systems which have inter-assay variability (151). It is important to note that the correct manner of use is as a monitoring device and not for diagnostic or treatment recommendations. Single values give only one point of reference, but sequential data can be an effective tool in the prevention of cardiovascular disease. This model and the algorithm are based on theoretical calculations. No clinical trial or original human data is included in the calculations. The algorithm is an adjunctive device for the evaluation of possible cardiovascular disease. Such a tool could be added to the clinical management of every individual. Additional data will need to be assessed with clinical studies.

## 6 Conclusion

Sequential data from this algorithm could act as a proxy for the process of *in situ* aging in cardiovascular tissue. The calculations can be done rapidly using the algorithm and 4 commonly used blood biomarkers. Combining presently available laboratory tests with dedicated software provides the clinician an inexpensive noninvasive tool to monitor the development of cardiovascular disease specific for each individual. It could predict individual cardiovascular changes and allow proactive management. This noninvasive monitoring system could be a significant advance in the prevention of cardiovascular disease. Once further investigation determines the usefulness of the algorithm, it should be made available to everyone to counter the global burden of heart disease.

## Data Availability

The original contributions presented in the study are included in the article/supplementary material, further inquiries can be directed to the corresponding author.
